# Anthropometric Variables and Somatotype of Young and Professional Male Basketball Players

**DOI:** 10.3390/sports6010009

**Published:** 2018-01-29

**Authors:** Karol Gryko, Anna Kopiczko, Kazimierz Mikołajec, Petr Stasny, Martin Musalek

**Affiliations:** 1 Department of Athletics and Team Sport Games, Józef Piłsudski University of Physical Education, Warszawa 00-968, Poland; gryczan@wp.pl; 2 Department of Anthropology and Health Promotion, Józef Piłsudski University of Physical Education, Warszawa 00-968, Poland; anna.kopiczko@awf.edu.pl; 3 Department of Theory and Practice of Sport, The Jerzy Kukuczka Academy of Physical Education, Katowice 40-065, Poland; k.mikolajec@awf.katowice.pl; 4 Faculty of Physical Education and Sport, Charles University, 162 52 Prague, Czech Republic; musalek@ftvs.cuni.cz

**Keywords:** maturation, elite sport, playing position, body composition, youth athletes, talent selection

## Abstract

Background: Determining somatic models and profiles in young athletes has recently become a fundamental element in selecting basketball playing positions. The aim of this study was to assess the relationship between the body build of young and adult elite male basketball players at different playing positions. Methods: Participants consisted of 35 young (age: 14.09 ± 0.30 years, *n* = 35) and 35 adult professional basketball players (age: 24.45 ± 5.40 years, *n* = 35) competing in elite leagues. The anthropometric characteristics assessed included body mass, body height, skinfolds, somatotypes, girths, and breadths. Results: The centers in both age groups were significantly taller and heavier (*p* < 0.001) compared to forwards and guards. The greatest difference between categories were in the guards’ personal height (from 169.36 to 186.68 = 17.32 cm). The guards from the professional team were closest in height to the forwards (difference = 7.17 cm) compared to young players where the difference between guards and forwards was 13.23 cm. Young competitors were more ectomorphic (2.12-3.75-4.17), while professional players were more mesomorphic (2.26-4.57-3.04). Significant criteria for center selection at professional level seems to be personal height and arm span ratio. Conclusions: The results indicate that the selection for basketball playing positions should include the analysis of body height and mass, shoulder breadth, humerus breadth, femur breadth and specifically for centers the difference between personal the height and arm span.

## 1. Introduction

Performance in basketball depends on many factors, with the most important one being players’ somatic build, as well as technical, tactical, motor, physiological, and psychological preparation. A basketball coach must supervise balanced development of players, i.e., physique, visual and motor coordination improvement and development of necessary motor abilities, considering evolutionary processes connected with the pace of growth and maturation of players [1,2,3]. In basketball, an individualized approach and making anthropometric diagnoses are basic elements of the selection process and of developing a long-term sports career.

Anthropometric measurements, determination of somatic build models, and somatic profiles have recently become fundamental research areas for sports training specialists [4,5,6,7,8]. Somatic profiles of basketball players have been widely recognized as a crucial factor in the selection process and as a performance predictor [5,9,10,11]. Anthropometric characteristics, such as body fat, skinfold thickness, body height, arm span, and body circumferences, were determined to be principal components in elite basketball players; therefore, they are often regarded as indicators of the level of play [8].

Previous analyses of somatic characteristics in basketball players indicate that body measurements are essential in the general selection process and in assigning playing positions [12]. Moreover, somatic parameters have an impact on players performance in condition tests [13]. Tests on both young and professional players revealed that individuals who were taller in stature, had more mesomorph component, and had longer limbs obtained higher scores regarding efficiency on the court and achieved better physiological parameters [14]. The crucial component in the process of assigning specific playing positions is body height [4], in which the tallest players are selected as centers (close to the basket), and those of shorter stature as guards (on the perimeter, further away from the basket) [5,15]. Additionally, the competitors playing in different positions also revealed differences in body girths (thigh, calf, arm, and forearm girths) between players [16].

Somatotype, defined as the description of such morphological components as endomorph, mesomorph and ectomorph, is another valuable tool for the accurate assessment of somatic parameters needed in a given sport [17]. Popovic et al. [18] observed that male basketball players are likely to display a mesomorph somatotype, but there are also professional players from top teams with mixed and balanced somatotypes. Moreover, the somatotype and other anthropometric variables might be specific to geographical region, especially during growth and maturation [19]. 

Considering the current state of knowledge in this field, it might be beneficial to examine breadth- and circumference-related aspects of body build. Moreover, there is lack of previous studies comparing the anthropometrics in young and senior elite basketball players. Therefore, the aim of this study was to compare body fat, length parameters, girths, circumferences, somatotypes and breadth-related measurements between players of different positions on young and adult male elite basketball teams. Furthermore, this study examined the relationship or specificity in selected anthropometric characteristics and basketball playing position.

## 2. Materials and Methods

Anthropometric measurements were assessed by experienced anthropometric technician in optimal climatic conditions in accordance with standards set by the International Society for the Advancement of Kinanthropometry (ISAK) [20]. The following variables were measured: age; basketball experience; body mass, body height and arm span (GPM anthropometer, Siber Hegner, Zurich, Switzerland); relaxed arm girths; flexed arm girth; calf girths (Holtain anthropometric tape, Crymych, UK); shoulder breadth; humerus breadth; femur breadth (GPM big and small spreading caliper, Zurich, Switzerland); and skinfolds from the triceps, subscapular, biceps, iliac crest, supraspinal, abdominal, and medial calf (Harpenden Skinfold Caliper, British Indicators, West Sussex, UK).

Body mass and body fat (BF) percentages were determined with the Bioelectrical Impedance Analysis (BIA) using the Tanita BC-418 device (Amsterdam, The Netherlands). Somatotype was calculated according to the Heath-Carter method [21] using the Somatotype 1.2.6 computer program (MER Goulding Software Development, Geeveston, Australia).

The study included 70 male basketball players from two different age categories (young and adult, Table 1). The first group consisted of young elite basketball players (*n* = 35) from the Mazovia regional team (age: 14.09 ± 0.30 years) that qualified for the 2014–2016 Polish Championships of Regional Teams. The team members are the best players selected from sports clubs in the Mazovia Region who are medalists from the Polish Youth Championships. The second group (*n* = 35) consisted of professional adult basketball players (age: 24.45 ± 5.40 years) competing in the highest-level league in Poland. The playing position of the players was determined by their common match nomination in regional team or professional club.

Prior to the commencement of the study, all of the participants were informed about the study’s aims and conduct, as well as about the possibility of resigning from research participation without providing any causes at any time. An informed consent provided by the participants or their legal representatives signature (if age below 18 years) was the study inclusion criterion, whereas contraindications for being subjected to anthropometric measurement procedures or bioelectrical impedance analysis were the exclusion criteria. The research was conducted in accordance with approval from the Ethics Committee for Scientific Research of the University of Physical Education in Warsaw, and the study was completed according to the rules and regulations of the Declaration of Helsinki [22].

**Figure 1:**
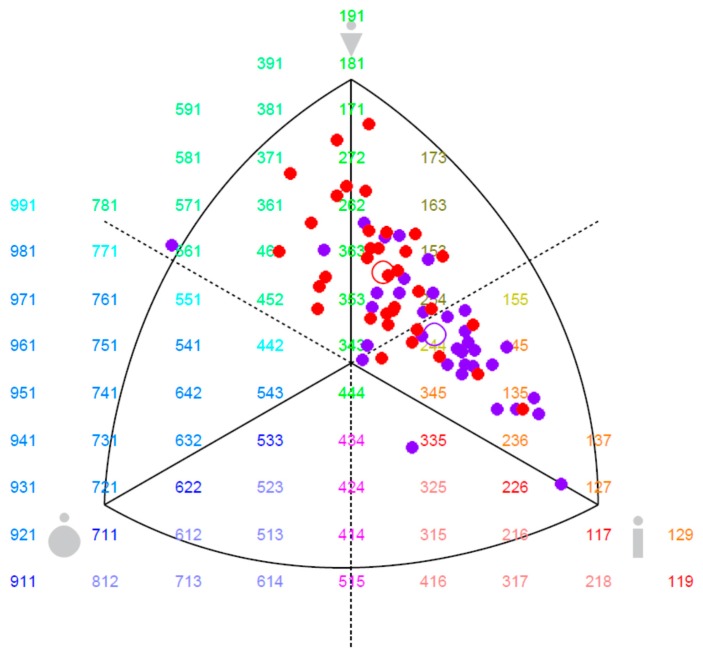
Somatochart of the examined basketball players. The circle is the mean profile of each group: • Adult professional basketball players, • Young basketball players.

All statistical analyses were performed in STATISTICA version 12 (StatSoft, Inc., Tulsa, OK, USA). The means, standard deviations (SD), and maximum and minimum values were used for group descriptions (Table 1), and somatotype values were expressed in a somatochart for both groups (Figure 1). The Shapiro–Wilk test was applied to examine the data normality distribution. One-way ANOVA (post-hoc Tukey tests, for equal sample sizes, with *p* < 0.01, Hays ω^2^ < 0.08 considered significant) was employed to assess the significance of differences in values referring to anthropometric and somatic features between young and professional groups of basketball players. The MANOVA (post-hoc Tukey tests, for unequal sample sizes, with *p* < 0.05, Hays ω^2^ < 0.09, considered significant) was used to show significant differences in parameters describing young and professional basketball players in respective positions. If appropriate, the Kruskal–Wallis non-parametric ANOVA was used for selected parameters. For ANOVA analyses, the players were divided into three groups according to playing positions: guards, forwards, and centers.

To determine the strength of the association between playing positions and anthropometric variables (nominal-by-interval variable), the Hay ω was used. Hay ω from 0.10–0.30 was thought to represent a weak association; coefficient from 0.30–0.50 was considered a moderate association; and coefficient of and greater than 0.50 was considered a strong association [23]. Effect size was calculated as Cohen f (Small effect: <0.10; medium effect: 0.10–0.40; large effect: <0.40) [24,25]. Significant differences and correlations was assumed at *p* < 0.05. 

## 3. Results

The Shapiro-Wilk test revealed no grounds for rejecting the hypothesis of normality in both groups without specification of playing position, and further in body height, body mass, fat percentage, breadth parameters and somototypes if considering players position. Anthropometric characteristics of young and adult male players revealed that young players demonstrated significantly (*p* < 0.001) lower values of body height, arm span, body mass, (body mass index) BMI, body fat (BF), shoulder breadth, humerus breadth and femur breadth (*p* < 0.01), see Table 1. Furthermore, younger players had significantly (*p* < 0.001) lower values of girth parameters: relaxed arm girth, flexed arm girth, calf girth and subscapular skinfold (Table 1). In addition, a significantly (*p* < 0.01) lower percentage of the mesomorphic component and higher percentage of the ectomorphic component were noted in young basketball players (Table 1 and Figure 1). Young as well as adult basketball players had wider arm span than personal height.

When considering the absolute differences in personal height between young and professional adult male basketball players regarding to players position, we revealed that increments were not proportionate. The greatest difference in personal height was identified in guards (from 169.36 to 186.68 cm, a difference of 17.32 cm). Guards and forwards from the adult professional team were close in height (difference = 7.17 cm), whereas guards and forwards on the young team were different in height (difference = 13.23 cm). In conformity with results regarding personal height, in young players, the arm span of 146 cm in centers was significantly greater (F_2,33_ = 32.89, *p* < 0.001, ω^2^ = 0.23) compared to guards and forwards (147 cm), and guards had the shortest arm span. Adult centers had a significantly wider arm span (F_2,33_ = 22.26, *p* < 0.01, Hays ω^2^ = 0.19) compared to guards and forwards. Professional players centers had significantly greater difference between personal height and arm span (F_2,33_ = 3.89, *p* < 0.05, ω^2^ = 0.14) compared to guards and forwards, which seems to be a significant factor for selection of center position: average difference between personal height and arm span: center = 6.86 cm; guards = 3.56 cm; forwards = 2.42 cm, respectively.

Guards in both categories had significantly narrower breadth of humerus epicondyle (young players, F_2,33_ = 24.12, *p* < 0.001, ω^2^ = 0.19; professional players: F_2,33_ = 13.22, *p* < 0.001, ω^2^ = 0.15) and femur epicondyle (young players, F_2,33_ = 45.12, *p* < 0.001, ω^2^ = 0.21; professional players, F_2,33_ = 14.78, *p* < 0.001, ω^2^ = 0.17) than centers. Additionally, young guards had lower shoulder breadth values (F_2,33_ = 130.9, *p* < 0.001, ω^2^ = 0.48) compared to forwards and centers (Table 2). The endomorphic component was greater in the centers than in the forwards (F_2,33_ = 26.12, *p* < 0.001, ω^2^ = 0.21) among young players. The ectomorphic component was significantly (F_2,33_ = 34.92, *p* < 0.001, ω^2^ = 0.27) more prevalent in the forwards than in the guards in young players (Table 2).

Based on anthropometric measurements, three somatic types were determined in basketball players from both groups. The mean somatotype of young players was characterized by the following code: 2.12-3.75-4.17, indicating that the average young player had an ecto-mesomorphic body build. The mean somatotype of professional players was defined by the following code: 2.26-4.57-3.04, meaning that the adult players were more meso-ectomorphic (Figure 1). 

Since a portion of measured skinfolds expressed non-parametric characteristics, we used Kruskal-Wallis non-parametric ANOVA to analyze these cases. In young players, there were clear differences between players of different positions in three front trunk skinfolds (iliac, supraspinal, and abdominal) and in triceps and medial calf skinfolds. Centers had significantly greater skinfold measurements than forwards in triceps (Chi-Square H_2,31_ = 8.75, *p* = 0.03), calf (H_2,31_ = 7.94, *p* = 0.02), iliac (H_2,31_ = 6.70, *p* = 0.01), supraspinal (H_2,31_ = 5.71, *p* = 0.01), and abdominal (H_2,31_ = 4.32, *p* = 0.03). On the other hand, for professional players, there were no significant differences in skinfold values among different player positions. 

Table 3 illustrates the correlations for the calculated anthropometric indices and playing positions (guards, forwards, centers) for each of the groups and as a combined dataset. In the group of young basketball players, significant strong positive correlations were found in body height (with large effect size), body mass, and shoulder breadth, while significant moderate positive correlations were noted between for all other parameters except arm span.

For professional basketball players, there were significant positive correlations between body mass (with large effect size), body height, flexed arm girth and playing position. Moreover, moderate positive correlations to playing position were noted in arm span, calf girth, relaxed arm girth, shoulder breath, humerus breath, femur breath, and BMI. 

## 4. Discussion

The main finding of this study is that the adult male professional players are more similar regarding playing position in somatic parameters than young male players. The weight and body height are the main selective parameters in both young and professional players, however the strength of this relationship is decreased in professional players for body height. The centers are taller and heavier than forward and guards, while basic somatic features, such as body mass and height, in the examined young and professional basketball players were similar to those observed in previous studies on elite players in Poland and abroad [12,26]. Our results showed that strong correlations were found between body height, body mass, shoulder breadth, and playing positions in young players. For adult players, there were strong correlations between playing positions and body mass, body height, and flexed arm girth.

Although young male players had most of anthropometric results associated with playing position (e.g., height), there was no significant associating between playing position and arm span (Table 3). On the other hand, professional players had moderate association between playing position and arm span. Because the arm span was previously associated with professional draft status [11,27] and professional centers have the largest arm span, the young players with largest arm span should be preferred on the centers position.

The present study revealed significantly smaller basic body parameters, such as body mass and height, in young male players. Analogous differences were found in breadth-related skeleton features (significantly lower values of arm span, shoulder breadth, humerus breadth and femur breadth were noted in young basketball players). These findings might amend the knowledge about ontogenetic variability of morphological and structural capabilities of basketball players in various stages of training. A previous study [28] reported the differences in body build between young (similar age as our group) and cadet age in measurements of epiphysis diameters, body fat, tibia length, femur and trunk length, while indicating the importance of proximal bone parts development.

Our findings can be referred to the results obtained by Abdelkrim [12], where Tunisian male basketball players demonstrated mean body heights between 192.0 cm and 198.4 cm (U-18: 192.0 ± 7.3 cm; U-20: 199.2 ± 7.3 cm; seniors: 198.4 ± 6.2 cm). Their mean body mass was between 83.7 kg and 91.5 kg (U-18: 83.7 ± 8.2 kg; U-20: 91.4 ± 8.3 kg; seniors: 91.5 ± 7.2 kg). Our research revealed greater differences in body height and mass in both groups, which might be a result of low age in our young group. Similar to the research on Tunisian basketball players [12] and Spanish players from different professional leagues [8], our investigation showed that, regardless of age, the centers were the tallest of all the players. These similar studies [8,12] and our study revealed that the mean body height of the centers was almost 200 cm. The centers also had the highest body mass compared to players from other positions. Another study conducted at the first National Collegiate Athletic Association (NCAA) male division showed higher centers (205.5 ± 6 cm) and forwards (198 ± 3.8) that our study [29]. The young centers in our study were smaller and had a shorted arm span than Australian plyers (U-16: height 195 ± 4 cm; arm span 199 ± 5 cm) [10].

A previous study [30] observed that performance outcomes like agility or vertical jumps in elite male players were not related to body fat content in basketball. Our study revealed that the centers from the young male group exhibited the highest values of measured skinfolds compared to the forwards. Notably, compared to forwards, the young centers demonstrated the highest values of abdominal, triceps, subscapular, suprailiacal and calf folds. Since the body fat and body fat distribution in our study were not associated with professional playing position, we suggest avoiding selecting young players to playing positions according to those parameters. 

The distribution of somatotypes in the group of young male players mainly covered the area of the somatogram close to ectomorphy, with the exception of two extreme cases (extreme ectomorph and endo-mesomorph). In adult professional players, the distribution close to mesomorphy prevailed. Our findings can be compared to a study by Martinez [31], who assessed somatotype profiles of Mexican Professional Basketball League players aged approximately 25 years. The mean value of endomorphic, mesomorphic, and ectomorphic components in that study was 2.94, 6.35, and 2.06, respectively. Our study showed that mean somatotype of professional players differed from that of Mexican competitors, i.e., the former group displayed lower values of endomorphic and mesomorphic components and higher values of ectomorphy than the latter. Regardless of their playing positions, Mexican competitors manifested extreme mesomorphic profiles, whereas professional players from our study were meso-ectomorphic what is more typical somatotype profile in elite collective sports [21,32,33]. 

Basketball training coaches claim that tasks performed by centers are fundamental in terms of offensive and defensive actions. Therefore, in elite teams, centers have specific body parameters that correlate with their roles on the court [7,34]. However, as a complex team sport, basketball requires proper a coach to not only train a professional team, but first and foremost, to identify and select children in the process of training. Thus, it is necessary to conduct further longitudinal research to determine useful body build characteristics and somatotypes in basketball players. 

One of the study’s limitation is the absence of biological age of young male players, where the greatest growth of Polish boys and greatest differences in biological age were observed between 12 and 15 years old [35]. The estimation of maturity offset by anthropometric measure was not possible with sufficient validity and reliability due to the extreme values of Polish elite basketball players [19,36]. Therefore, we can´t avoid that elite players were included in the observed team for their earlier maturation [37,38] or another bias. Another limitation is the absence of performance values of measured players, however these data have to be collected during ongoing longitudinal and further research rather than from retrospective statistic. 

## 5. Conclusions

The results of this study indicate that anthropometric assessment of body build, as well as somatotype analysis, may be key factors in the process of talent identification in basketball. It should be highlighted that the selection for basketball and specifically for playing positions should include the analysis of somatic build features such as body height and mass, shoulder breadth, flexed arm girth and arm span. According to specialists, basketball is a dynamic team sport and, therefore, the determination of body build profiles may become a key factor in assessing players’ capabilities in regard to their fitness levels and efficiency during performance. It seems that somatic parameter differences between player positions in young male players does not play a key role in adult players. In male adults, there are somatic predispositions for centers (such as the height, weight, arm span and girths), while the body build of forwards tends to be similar to that of the centers. The position with the lowest requirement for body size is the guard. Coaches should not pay attention to the body fat and body fat distribution to select players to their playing position of young male players. On the other hand the height, weight and arm span should be considered for such selection. 

## Figures and Tables

**Table 1:** Characteristics of young and professional male basketball players and differences between both groups.

Variable	Young Players (*n* = 35)		Adult Professional Players (*n* = 35)	
	Mean ± SD	Range	Mean ± SD	Range
Age (years)	14.09 ± 0.30	13.37–14.47	24.45 ± 5.40	18.45–36.83
Basketball experience (years)	3.73 ± 1.24	1.0–7.0	13.43 ± 3.53	8.0–22.0
Body mass (kg)	64.98 ± 10.70 ^†^	43.6–99.7	90.23 ± 10.50	72.6–116.6
Body height (cm)	179.22 ± 8.41 ^†^	158.8–194.1	193.44 ± 8.07	174.3–219.0
Arm span (cm)	183.09 ± 8.15 ^†^	165.5–196.6	197.78 ± 9.17	179.1–223.0
Body mass index (BMI)	20.12 ± 2.16 ^†^	17.0–28.2	24.0 ± 1.81	19.9–28.1
Body fat (%)	11.0 ± 3.79 ^†^	4.3–22.8	14.01 ± 3.06	8.7–22.60
Triceps skinfold (mm)	8.64 ± 3.23	5.0–24.0	7.56 ± 2.38	3.3–13.3
Subscapular skinfold (mm)	7.51 ± 3.03 ^†^	5.0–23.2	10.18 ± 2.15	6.5–18.2
Biceps skinfold (mm)	4.46 ± 1.59	3.0–10.0	4.78 ± 1.64	3.07–9.73
Iliac crest skinfold (mm)	10.18 ± 4.52	6.2–25.5	11.79 ± 4.76	6.23–27.2
Supraspinal skinfold (mm)	7.02 ± 3.12	4.1–15.5	8.30 ± 2.45	4.7–14.9
Abdominal skinfold (mm)	10.2 ± 5.98	4.3–29.0	9.91 ± 4.81	5.0–28.3
Medial calf skinfold (mm)	9.07 ± 4.08	4.5–26.0	7.64 ± 2.96	3.53–17.0
Relaxed arm girth (cm)	25.60 ± 2.36 ^†^	20.5–32.0	31.37 ± 2.03	28.2–36.0
Flexed arm girth (cm)	27.89 ± 2.38 ^†^	22.4–34.4	34.80 ± 2.23	30.2–38.0
Calf girth (cm)	35.73 ± 2.83 ^†^	30.0–46.0	39.63 ± 2.45	35.0–45.0
Shoulder breadth (cm)	39.04 ± 1.83 ^†^	35.4–43.1	42.90 ± 1.72	39.8–47.2
Humerus breadth (cm)	7.09 ± 0.43 ^†^	6.0–8.0	7.59 ± 0.51	6.7–8.9
Femur breadth (cm)	9.91 ± 0.43 *	9.0–10.7	10.39 ± 0.84	9.0–12.00
Endomorphy	2.12 ± 0.81	1.16–5.57	2.26 ± 0.59	1.19–3.66
Mesomorphy	3.75 ± 1.01 *	1.23–5.88	4.57 ± 1.07	2.31–6.95
Ectomorphy	4.17 ± 1.08 ^†^	1.18–6.36	3.04 ± 0.89	1.22–5.38

* Significantly different from adult professional basketball players (*p* < 0.01); ^†^ Significantly different from adult professional basketball players (*p* < 0.001).

**Table 2:** Baseline characteristics of young and professional basketball male players in relation to their playing positions.

Variable	Guards		Forwards		Centers	
	Young (*n* = 12)	Adult Professional (*n* = 12)	Young (*n* = 11)	Adult Professional (*n* = 11)	Young (*n* = 12)	Adult Professional (*n* = 12)
Age	14.14 ± 0.31	23.94 ± 5.17	14.03 ± 0.33	24.37 ± 5.13	14.10 ± 0.28	25.04 ± 6.24
Body mass (kg)	^C^ 57.10 ± 6.64 ^†^	^C^ 81.08 ± 4.61	^C^ 63.71 ± 6.67 ^†^	^C^ 89.25 ± 8.55	^G,F^ 74.02 ± 10.51 ^†^	^G,F^ 100.29 ± 7.10
Body height (cm)	^C^ 169.36 ± 5.16 ^†^	^C^ 186. 68 ± 5.9	^C^ 182.59 ± 3.81 ^†^	^C^ 193.85 ± 4.39 ^†^	^G,F^ 185.98 ± 3.39 ^†^	^G,F^ 199.83 ± 7.37
Arm span (cm)	^C^ F174.44 ± 5.98 ^†^	^C^ 190.23 ± 4.52	^G^ 184.90 ± 3.11 ^†^	^C^ 196.28 ± 5.82	^G^ 190.08 ± 4.76 ^†^	^G,F^ 206.70 ± 7.58
Body mass index	19.88 ± 1.51 ^†^	23.06 ± 0.86	19.11 ± 1.46 ^†^	23.75 ± 2.09	21.28 ± 2.78 ^†^	25.16 ± 1.72
Body fat (%)	11.07 ± 3.18	13.21 ± 1.93	9.53 ± 3.09	13.28 ± 3.13	12.27 ± 4.65	15.47 ± 3.58
Triceps SF (mm)	8.29 ± 1.04	8.01 ± 2.58	^C^ 7.17 ± 2.02	6.48 ± 1.8	^F^ 10.33 ± 4.7	8.1 ±2.49
Subscapular SF (mm)	6.88 ± 1.45	9.82 ± 1.46	^C^ 6.61 ±1.07	9.51 ± 2.08	^F^ 8.97 ± 4.64	11.14 ± 2.57
Biceps SF (mm)	4.21 ± 1.0	5.31 ± 2.35	3.85 ± 1.07	4.44 ± 1.26	5.27 ± 2.15	4.58 ± 0.96
Iliac skinfold (mm)	8.94 ± 2.63	12.16 ± 4.5	^C^ 8.06 ± 1.12	10.73 ± 4.03	^F^ 3.37 ± 6.16	12.38 ± 5.77
Supraspinal SF (mm)	6.25 ± 1.99	8.11 ± 2.46	^C^ 5.48 ± 1.3	8.34 ± 2.02	^F^ 9.21 ± 4.05	8.46 ± 2.96
Abdominal SF (mm)	9.22 ± 4.78	9.43 ± 4.4	^C^ 7.27 ± 1.43	9.28 ± 3.18	^F^ 13.87 ± 7.84	10.96 ± 6.42
Medial calf SF (mm)	8.37 ± 2.18	7.31 ± 2.47	^C^ 6.95 ± 2.03	7.06 ± 2.73	^F^ 11.72 ± 5.5	8.51 ± 3.6
Relaxed arm girth (cm)	24.82 ± 1.81 ^†^	30.54 ± 1.64	25.04 ± 2.34 ^†^	30.64 ± 2.5	26.88 ± 2.49 ^†^	32.88 ± 0.79
Flexed arm girth (cm)	27.25 ± 2.22 ^†^	^C^ 34.04 ± 1.67	27.0 ± 1.7 ^†^	^C^ 33.68 ± 2.62	29.35 ± 2.53 ^†^	^F,G^ 36.57 ± 1.0
Calf girth (cm)	34.38 ± 1.84 ^†^	^C^ 38.07 ± 1.86	35.74 ± 1.89 ^†^	39.8 ± 2.71	37.09 ± 3.75 ^†^	^F^ 41.04 ± 1.86
Shoulder breadth (cm)	^C,F^ 37.35 ± 1.14 ^†^	41.89 ± 1.48	^G^ 39.38 ± 1.46 ^†^	42.69 ± 1.15	^G^ 40.43 ± 1.33 ^†^	44.09 ± 1.75
Humerus breadth (cm)	^C^ 6.83 ± 0.37 ^†^	^C^ 7.36 ± 0.42	7.07 ± 0.37	7.54 ± 0.41	^G^ 7.36 ± 0.39 *	^G^ 7.88 ± 0.56 *
Femur breadth (cm)	^C^ 9.65 ± 0.41	^C^ 9.98 ± 0.66	9.97 ± 0.42	10.7 ± 0.82	^G^ 10.12 ± 0.35	^G^ 10.52 ± 0.92
Endomorphy	2.09 ± 0.42	2.34 ± 0.49	^C^ 1.67 ± 0.37	2.07 ± 0.54	^F^ 2.56 ± 1.14	2.34 ± 0.72
Mesomorphy	4.34 ± 0.68	4.62 ± 0.9	3.23 ± 0.83 *	4.51 ± 1.42	3.64 ± 1.17	4.59 ± 0.94
Ectomorphy	^F^ 3.68 ± 0.7	3.0 ± 0.66	^G^ 4.94 ± 0.81 *	3.22 ± 1.06	3.96 ± 1.26	2.92 ± 0.96

^†^ Significantly different between young and adult professional at *p* < 0.01, * Significantly different between from and adult professional at *p* < 0.05, ^C^ Significantly different from centers in the same age group at *p* < 0.05, ^F^ Significantly different from forwards in the same age group at *p* < 0.05, ^G^ Significantly different between guards in the same age group at *p* < 0.05, SF = skinfold.

**Table 3:** The strength of association for the calculated anthropometric variables and playing positions (guards, forwards, centers) for each group of male basketball players as a combined dataset.

Variable	Young Players (*n* = 35)	Effect Size Young	Adult Players (*n* = 35)	Effect Size Adult
Body mass (kg)	0.62 **	0.31 ^†^	0.75 **	0.64 ^††^
Body height (cm)	0.86 **	1.42 ^††^	0.66 **	0.38 ^†^
Arm span	0.17	0.02	0.38 *	0.09
Triceps skinfold (mm)	0.33 *	0.06	0.2	0.02
Subscapular skinfold (mm)	0.25	0.03	0.25	0.03
Biceps skinfold (mm)	0.31 *	0.06	0.21	0.2
Iliac crest skinfold (mm)	0.47 *	0.14 ^†^	0.02	<0.01
Supraspinal skinfold (mm)	0.47 *	0.14 ^†^	0.09	<0.01
Abdominal skinfold (mm)	0.41 *	0.10	0.09	<0.01
Medial calf skinfold (mm)	0.44 *	0.12 ^†^	0.10	<0.01
Relaxed arm girth (cm)	0.32 *	0.06	0.49 *	0.15 ^†^
Flexed arm girth (cm)	0.39 *	0.09	0.54 **	0.20 ^†^
Calf girth (cm)	0.32 *	0.06	0.46 *	0.13 ^†^
Shoulder breadth (cm)	0.69 **	0.45 ^††^	0.49 *	0.15 ^†^
Humerus breadth (cm)	0.46 *	0.13 ^†^	0.36 *	0.07
Femur breadth (cm)	0.41 *	0.10	0.32 *	0.04
Endomorphy	0.4 *	0.10	0.03	<0.01
Mesomorphy	0.4 *	0.10	0.02	<0.01
Ectomorphy	0.46 *	0.13	0.09	<0.01
Body mass index (BMI)	0.35 *	0.06	0.44 *	0.12 ^†^
Body Fat (%)	0.35 *	0.06	0.37	0.08

* Moderate association: 0.30–0.50; ** strong association: >0.50; ^†^ medium effect size: 0.10–0.40; ^††^ strong effect size: >0.40.
